# A novel nanoparticle-based co-delivery system of *Fritillaria persica L*. extract and gemcitabine for inhibiting breast cancer progression

**DOI:** 10.3762/bjnano.17.96

**Published:** 2026-10-15

**Authors:** Reyhaneh Arefimehr, Mehrad Zibaei Moghadam, Negar Jamshidi, Nazanin Jamshidi

**Affiliations:** 1 Department of Biology,Central Tehran Branch, Islamic Azad University, Tehran, Iran; 2 Kimia Andisheh Gene Pouyan Medical and Molecular Laboratory Research Co, Tehran, Iran

**Keywords:** apoptosis, breast cancer, *Fritillaria persica*, gemcitabine, proliferation

## Abstract

Breast cancer remains one of the leading causes of cancer-related mortality worldwide, highlighting the urgent need for more effective and less toxic therapeutic strategies. In the present study, a novel nanotherapeutic platform composed of aqueous *Fritillaria persica L*. (FP) extract and gemcitabine (Gem)-loaded chitosan (CS) nanoparticles (FP/Gem/CS-NPs) was successfully developed and evaluated against MCF-7 and MDA-MB-231 breast cancer cell lines. Physicochemical characterization demonstrated the successful fabrication of spherical FP/Gem/CS-NPs with a mean hydrodynamic diameter of approximately 69 nm, a polydispersity index (PDI) of 0.500, and a zeta potential of −16.2 mV. The nanoparticles demonstrated high entrapment efficiency (78.41%) and sustained drug release behavior, with nearly 50% of Gem released over 72 h, compared with the rapid burst release of free gemcitabine. Biological evaluations revealed that FP/Gem/CS-NPs exerted significantly enhanced anticancer activity compared with free Gem or FP extract alone. MTT assays demonstrated strong dose- and time-dependent cytotoxicity, with markedly reduced IC_50_ values in both MCF-7 and MDA-MB-231 cells following nanoparticle treatment. Flow cytometry and caspase-3/7 analyses confirmed substantial apoptosis induction, particularly in the highly aggressive MDA-MB-231 cells. Gene expression analysis further demonstrated significant upregulation of pro-apoptotic BAX and cell cycle regulator P21, alongside downregulation of anti-apoptotic BCL-2, indicating activation of mitochondrial apoptotic pathways. Moreover, NRF2 expression was elevated, suggesting modulation of oxidative stress responses. Cell cycle analysis revealed prominent G2/M phase arrest in treated cells. Collectively, these findings indicate that FP/Gem/CS-NPs represent a promising synergistic nanotherapeutic system capable of enhancing gemcitabine efficacy while modulating apoptosis, oxidative stress, and cell cycle pathways in breast cancer cells.

## Introduction

Breast cancer remains a major global health burden, with millions of new cases diagnosed and hundreds of thousands of deaths reported annually worldwide [1]. Despite substantial advances in diagnostic and therapeutic strategies, breast cancer continues to pose significant challenges due to its heterogeneity, recurrence, metastasis, and treatment resistance. These limitations underscore the urgent need to develop innovative and more effective therapeutic approaches aimed at improving clinical outcomes and patients’ quality of life [2–3].

Gemcitabine (Gem), a deoxycytidine analog, is a potent chemotherapeutic agent that inhibits DNA synthesis and induces apoptosis in rapidly proliferating cancer cells [4]. It has been widely used in the treatment of several malignancies, including pancreatic, lung, and bladder cancers [5–6]. Although Gem is not routinely used as a first-line therapy for breast cancer, it has demonstrated promising therapeutic potential in metastatic and triple-negative breast cancer, particularly in cases where conventional regimens fail due to chemoresistance [7–8]. In these settings, Gem is frequently combined with agents such as paclitaxel, carboplatin, or trastuzumab to enhance therapeutic efficacy and overcome resistance mechanisms [9]. However, despite its anticancer activity, the clinical application of Gem is often limited by systemic toxicity, rapid metabolism, poor bioavailability, and the development of drug resistance [10–11].

To overcome these limitations, increasing attention has been directed toward nanoparticle-based drug delivery systems and natural bioactive compounds as alternative or adjunct therapeutic strategies [12–15]. Among various nanocarriers, chitosan has attracted considerable interest because of its favorable physicochemical and biological properties. Chitosan is a natural biopolymer derived from the deacetylation of chitin found in crustacean exoskeletons and is characterized by excellent biocompatibility, biodegradability, mucoadhesive properties, and gel-forming ability [16–17]. These features make chitosan particularly suitable for biomedical and pharmaceutical applications, especially in cancer drug delivery systems.

Chitosan nanoparticles (CS-NPs) have emerged as efficient nanocarriers capable of improving drug solubility, stability, controlled release, and targeted delivery [18–21]. Previous studies have demonstrated that CS-NPs can enhance intracellular drug uptake, improve therapeutic efficacy, inhibit tumor progression, and promote apoptosis through the efficient encapsulation and delivery of anticancer agents [22–23]. Consequently, CS-NPs are considered promising platforms for reducing the adverse effects of chemotherapy while enhancing antitumor activity.

Recently, the incorporation of plant-derived compounds into nanoparticle systems has gained attention as part of the growing field of green nanomedicine [24]. *Fritillaria persica L.* (FP), a flowering medicinal plant traditionally used for the treatment of respiratory and inflammatory disorders, has shown considerable pharmacological potential [25–26]. Bioactive constituents of FP, including alkaloids, flavonoids, and glycosides, have been reported to possess antioxidant, anti-inflammatory, and anticancer activities. Emerging evidence also suggests that FP extracts may exert cytotoxic effects and induce apoptosis in different cancer cell lines [27], supporting their potential application as complementary therapeutic agents in cancer treatment.

In the present experimental study, we developed a novel co-delivery system consisting of aqueous FP extract and Gem-loaded chitosan nanoparticles (FP/Gem/CS-NPs) to improve the therapeutic efficacy of Gem against breast cancer cells. This approach aimed to exploit the biocompatibility and controlled-release properties of chitosan while enhancing the anticancer effects of Gem through synergistic interactions with FP bioactive compounds. Specifically, we investigated the effects of FP/Gem/CS-NPs on the expression of apoptosis-related genes, including BAX, BCL-2, P21, and NRF2, as well as on the metastatic behavior of MCF-7 and MDA-MB-231 breast cancer cell lines.

## Materials and Methods

### Materials

Gem was purchased from Sigma-Aldrich (St. Louis, MO, USA). The plant FP was obtained from the National Center for Genetic Resources, Iran (IBRC P1011066). Medium-molecular-weight chitosan was purchased from Sigma-Aldrich (Product No. 448877; 75–85% deacetylation; 200–800 cP viscosity of 1% w/v in 1% v/v acetic acid). Sodium tripolyphosphate and glacial acetic acid were also procured from Sigma-Aldrich. The human breast carcinoma cell lines MCF-7 and MDA-MB-231 were obtained from the Pasteur Institute (Tehran, Iran). Dulbecco’s Modified Eagle Medium (DMEM), fetal bovine serum (FBS), penicillin–streptomycin, and trypsin–EDTA were supplied by Gibco (Thermo Fisher Scientific, USA).

### Extraction of *Fritillaria persica L*

The plant FP was obtained from the National Center for Genetic Resources, Iran, under the accession code IBRC P1011066. The plant species was taxonomically identified and authenticated by a botanist, and a voucher specimen was deposited in a herbarium for future reference. FP bulbs were extracted to isolate bioactive compounds using the maceration method. The bulbs were first cleaned, air-dried, and ground into a fine powder. The powdered material was mixed with ethanol at a 1:10 (w/v) ratio in a sealed glass container and incubated at room temperature for 48 h in a shaking incubator to facilitate extraction. After incubation, the mixture was filtered through Whatman No. 1 filter paper to separate the liquid extract from the solid residues. The filtrate was concentrated using a rotary evaporator under reduced pressure to remove the solvent, yielding a thick crude extract. The resulting extract was stored in dark glass containers at 4 °C to preserve the stability and bioactivity of the phytochemicals until further use.

### Preparation and characterization of Gem/CS-NPs

Gem/CS-NPs were prepared using the ionic gelation technique with slight modifications [28]. Briefly, 17.56 mg of chitosan was dissolved in 10 mL of glacial acetic acid (1% v/v, pH 5.5). Separately, 7.4 mg of Gem (42% w/w) was dissolved in 1 mL of 0.1 M sodium hydroxide, mixed with 1 mL of tripolyphosphate solution (5.82 mg/mL), and added dropwise to the chitosan solution. The resulting mixture was stirred continuously for 60 min at room temperature and then centrifuged at 13,500 rpm for 20 min twice to remove unencapsulated Gem and excess tripolyphosphate solution. The pellets were resuspended in Milli-Q water, and the supernatant was collected for free drug quantification. The synthesized Gem/CS-NPs were characterized using various techniques, including ultraviolet spectroscopy, field-emission scanning electron microscopy (FESEM), and energy-dispersive X-ray analysis, and were subsequently stored at 4 °C for further experiments [29].

### Post-loading of *Fritillaria persica* extract onto Gem/CS-NPs (FP/Gem/CS-NPs)

The aqueous extract of *Fritillaria persica* (5 mg/mL) was added to the preformed Gem/CS-NP suspension and gently stirred for 1 h at room temperature to facilitate the association of the FP phytochemicals with the chitosan nanoparticle matrix through adsorption and non-covalent interactions. The resulting FP/Gem/CS-NPs were subsequently collected by centrifugation and washed with distilled water to remove unbound extract remaining in the aqueous phase. The purified nanoparticles were then stored at 4 °C until further physicochemical characterization and biological evaluation. No drying step was applied before biological evaluation to minimize possible changes in nanoparticle structure and drug release behavior.

### Characterization of nanoparticles

Dynamic light scattering (DLS, Malvern Zetasizer Nano ZS) was used to determine the average particle size, polydispersity index (PDI), and zeta potential. The morphology of the nanoparticles was analyzed using field-emission scanning electron microscopy (FESEM, Hitachi S-4800) after gold sputter coating. Ultraviolet–visible (UV–vis) spectroscopy (Shimadzu UV-1800) was used to confirm Gem loading. Fourier-transform infrared (FTIR) spectroscopy (Bruker Tensor 27) was performed to verify chemical interactions between chitosan, Gem, and FP extract. TEM analysis was not performed in this study due to technical limitations; therefore, nanoparticle morphology was evaluated using FESEM combined with DLS analysis.

### Entrapment efficiency and loading capacity

Entrapment efficiency (EE%) and loading capacity (LC%) of Gem in Gem/CS-NPs were determined according to Esnaashari and coworkers [28]. Following the synthesis of Gem/CS-NPs, the nanoparticle suspension was centrifuged to separate the free drug from the loaded nanoparticles. The recovered nanoparticles were subsequently dried to constant weight before determination of LC. The amount of unencapsulated Gem present in the supernatant was quantified at 268 nm using a Shimadzu UV-1800 UV–vis spectrophotometer. EE% and LC% were subsequently calculated using the following equations.


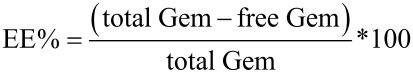



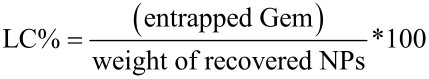


### Field-emission scanning electron microscopy

FESEM was employed to analyze the surface morphology of the synthesized FP/Gem/CS-NPs nanoparticles. A dilute suspension of FP/Gem/CS-NPs (0.1–0.5 mg/mL) was prepared in ethanol and sonicated (5–10 min) to ensure uniform dispersion. A small volume (5–10 μL) of the FP/Gem/CS-NP suspension was then drop-cast onto a clean silicon wafer, allowing it to dry thoroughly under ambient conditions. Once dried, the samples were coated with a thin gold layer (5–10 nm) to improve conductivity. Synthesized FP/Gem/CS-NPs were imaged using an FESEM instrument run at an accelerating voltage of 20 kV. Morphological features were documented at different magnifications, and ImageJ software was used for particle size analysis and distribution.

### Zeta potential measurement

The zeta potential of the FP/Gem/CS-NP formulation was measured using a Zetasizer Nano ZS (Malvern Instruments Ltd., UK) to assess the surface charge and colloidal stability of the nanoparticles. Before measurement, nanoparticle suspensions were diluted to an appropriate concentration in deionized water to avoid multiple scattering effects. Measurements were conducted at 25 °C using a clear disposable zeta cell with an effective voltage of 149 V and an attenuator setting of 7). Each sample was analyzed in triplicate, with 12 runs per sample, and the electrophoretic mobility was converted to zeta potential values using the Smoluchowski equation. The dispersant properties (refractive index = 1.330; viscosity = 0.8872 cP) were defined according to the instrument’s settings for water. The instrument’s software recorded and processed the zeta potential distribution, electrophoretic mobility, frequency shift, and phase plot. The mean zeta potential, standard deviation, and population distribution were automatically calculated, and all values are reported as the average of at least three independent measurements.

### In vitro drug release study

The in vitro release profile of free Gem and Gem loaded in FP/Gem/CS-NPs was evaluated using the dialysis bag diffusion method (molecular weight cutoff = 7000 Da) at predetermined time intervals. Briefly, free gemcitabine and FP/Gem/CS-NPs were separately dispersed in 3 mL of phosphate-buffered saline (PBS, pH 7.4) and transferred into dialysis bags. The dialysis bags were then immersed in the release medium and incubated at 37 °C under continuous shaking. At specific time points, 100 μL of the release medium was withdrawn and replaced with an equal volume of fresh PBS to maintain sink conditions. The amount of released Gem was quantified using a UV–vis spectrophotometer at 268 nm. Finally, the cumulative percentage of drug release was calculated according to the following equation.

### DPPH assay

The antioxidant activity of the treatments was evaluated using the 2,2-diphenyl-1-picrylhydrazyl (DPPH) radical scavenging assay, according to the manufacturer’s instructions provided with the Xantox commercial kit (Birjand, Iran). In brief, 250 μL of DPPH solution in ethanol was mixed with 5 µL of the test sample or 5 µL of the standard solution in microplate wells. The mixtures were gently shaken and incubated in the dark at room temperature for 15 min to allow for reaction completion. The absorbance of each sample was measured at 516 nm using a microplate spectrophotometer (INNO-M, South Korea), with Trolox serving as the reference antioxidant standard. The antioxidant capacity was expressed as micromoles of Trolox equivalents per liter.

### Cell culture

Human breast cancer cell lines, MCF-7 and MDA-MB-231, were obtained from the Genetic Resources Center Bank. The cells were cultured in Dulbecco’s Modified Eagle Medium (DMEM), which was supplemented with 10% fetal bovine serum, 100 U/mL penicillin, and 100 μg/mL streptomycin. Cells were incubated at 37 °C in a humid atmosphere with 5% CO_2_.

### MTT assay

The cytotoxicity of FP/Gem/CS-NPs was assessed using the 3-(4,5-dimethylthiazol-2-yl)-2,5-diphenyltetrazolium bromide (MTT) assay. Cells were cultured in 96-well plates and treated with a range of concentrations of the test formulations. After the treatment period, 20 μL of MTT solution (0.5 mg/mL) was added to each well, and the plates were incubated in the dark at 37 °C for 4 h. Following this incubation, the medium was carefully removed, and then, 150 μL of dimethyl sulfoxide was added to each well to dissolve the formazan crystals. Absorbance was measured at 570 nm using a microplate reader, and the half-maximal inhibitory concentration (IC_50_) was calculated from the dose-response curve.

### Caspase-3/7 activy

Caspase-3/7 activity in MCF-7 and MDA-MB-231 cells was evaluated using a commercial caspase activity assay kit (Kiazist, Iran) following a 24 h treatment with control and FP/Gem/CS-NP. After treatment, cells were harvested using trypsin-EDTA, washed twice with cold PBS, and lysed in the provided lysis buffer (50–100 μL per 1 × 10^6^ cells). The lysates were incubated on ice (10–15 min) and then centrifuged (13000 rpm, 10 min, at 4 °C), and the resulting supernatant was collected and mixed with an equal volume of 2× reaction buffer containing dithiothreitol. Next, the fluorogenic caspase-3/7-specific substrate (DEVD-AFC) was added to the mixtures. The reaction mixtures were incubated in the dark at 37 °C for 1 to 2 h. Following the kit protocol, fluorescence intensity was measured at 400 nm excitation and 505 nm emission using a microplate reader.

### Apoptosis

After treatment with various formulations, apoptotic cell death in MCF-7 and MDA-MB-231 cell lines was evaluated using a flow cytometer. Posttreatment, the cells were harvested using trypsin-EDTA, collected by centrifugation, and washed twice with PBS to remove residual enzymes. The cell pellets were resuspended in binding buffer, and the cell density was adjusted to 1–5 × 10^6^ cells/mL. Apoptosis was assessed using an Annexin V-based staining protocol, performed according to the manufacturer’s instructions. After staining, cells were washed to remove excess dye, centrifuged at 400*g* for 5 min, and gently resuspended in fresh binding buffer. Samples were analyzed using a flow cytometer, and the resulting data were processed using Flow-Jo software (version 10.8; BD Biosciences Ashland) [30].

### Cell cycle analysis

After treatment with various formulations, cell cycle analysis in MCF-7 and MDA-MB-231 cell lines was evaluated using a flow cytometer. Posttreatment, the cells were harvested using trypsin-EDTA, collected by centrifugation, and washed twice with PBS to remove residual enzymes. Cell cycle analysis in was assessed using a propidium iodide staining protocol, performed according to the manufacturer’s instructions. Samples were analyzed using a flow cytometer, and the resulting data were processed using Flow-Jo software (version 10.8; BD Biosciences Ashland) [30].

### Real-time PCR

According to the manufacturer’s instructions, total RNA was extracted from MCF-7 and MDA-MB-231 cells using RNX Plus solution (Sinacolon, Iran). Complementary DNA (cDNA) synthesis was performed using the PrimeScript RT Reagent Kit (TaKaRa, Japan) following the specified protocol. The BAX, BCL-2, P21, and NRF2 gene expression levels were quantified using Real Q Plus 2× Master Mix Green (Pars-Tous, Iran) and gene-specific primers. All reactions were conducted in triplicate for each experimental group. Relative gene expression was calculated using the ΔΔCt method, with normalization against a GAPDH. The sequences of the primers are listed in Table 1.

**Table 1:** Primer sequences used for gene expression analysis by qRT-PCR.

Gene symbol	Accession number	Primer sequences (5'–3')		PCR product length (bp)	*T*_m_ (°C)

GAPDH	NM_001289746.2	F	AAGGCTGTGGGCAAGGTCATC	248	58
		R	GCGTCAAAGGTGGAGGAGTGG		
BAX	NM_004324.4	F	ACAGGATGCGTCCACCAAGAAG	103	56
		R	AGTGTCCACGGCGGCAATCATC		
BCL-2	NM_000633.3	F	TTCGCCCTGTGGATGACTGAGT	127	54
		R	CCCAGGAGAAATCAAACAGAGGC		
P21	NM_001374511.1	F	TCTTGTACCCTTGTGCCTCG	176	59
		R	AGACAGGTCAGCCCTTGGAC		
NRF2	NM_001313903.2	F	TAGATCTTGGAGTTGCCCACAT	117	59
		R	ACGTAGCCGAAGAAACCTCA		

### Statistical analysis

Three independent experiments were conducted, and the data are presented as means ± standard deviation (SD). Statistical analyses were performed using Student’s t-test for comparisons between two groups, and two-way analysis of variance (ANOVA) for comparisons among multiple groups. The significance level of α = 0.05 was set, with *p* < 0.05 considered statistically significant. All analyses were conducted using GraphPad Prism (version 8).

## Results

### UV–vis spectroscopic characterization of FP/Gem/CS-NPs

**Figure 1:**
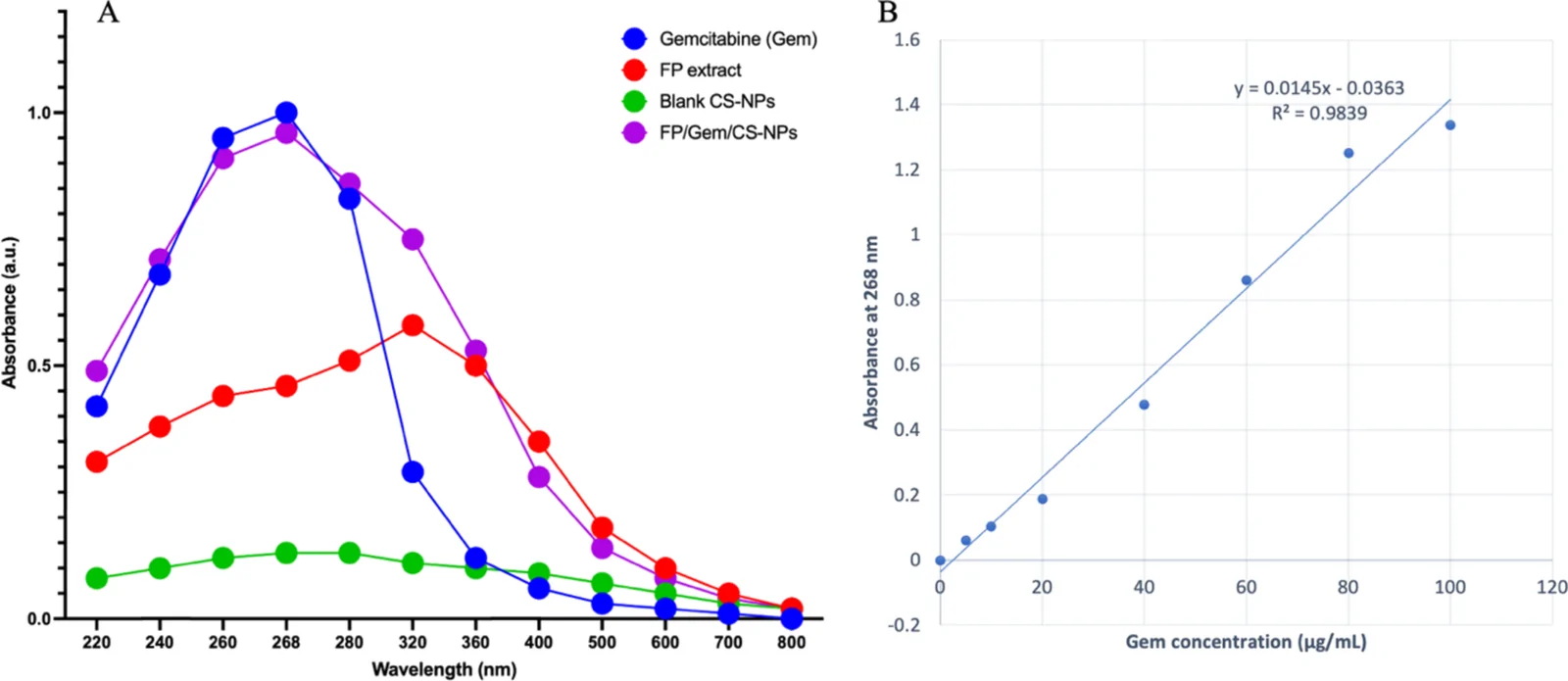
Characterization of Gem-loaded chitosan nanoparticles. (A) UV–vis absorption spectra of free gemcitabine (Gem), FP extract, blank CS-NPs, and FP/Gem/CS-NPs. (B) Standard calibration curve of Gem at 268 nm used for quantification of Gem during entrapment efficiency and in vitro release studies.

The UV–vis absorption spectra of free Gem, FP extract, blank chitosan nanoparticles (CS-NPs), and FP/Gem/CS-NPs were recorded over the wavelength range of 200–800 nm (Figure 1A). Free Gem exhibited a characteristic absorption peak at approximately 268 nm, whereas the FP extract showed a broad absorption band in the 300–340 nm region, which can be attributed to its phytochemical constituents. Blank CS-NPs displayed only weak absorbance without any distinct absorption peak. The spectrum of FP/Gem/CS-NPs retained the characteristic absorption features of both Gem and FP extract, indicating the successful incorporation of Gem and FP into the chitosan nanoparticle matrix. In addition, a standard calibration curve of Gem at 268 nm (Figure 1B) demonstrated excellent linearity and was used for quantitative determination of Gem during the entrapment efficiency and in vitro release studies.

### Entrapment efficiency and loading capacity

The EE% and LC% of Gem in FP/Gem/CS-NPs were quantified using UV–vis spectroscopy. The results demonstrated that the EE% of Gem was 78.41%. Furthermore, the LC% was determined to be 12.34%.

#### Morphological characterization

**Figure 2:**
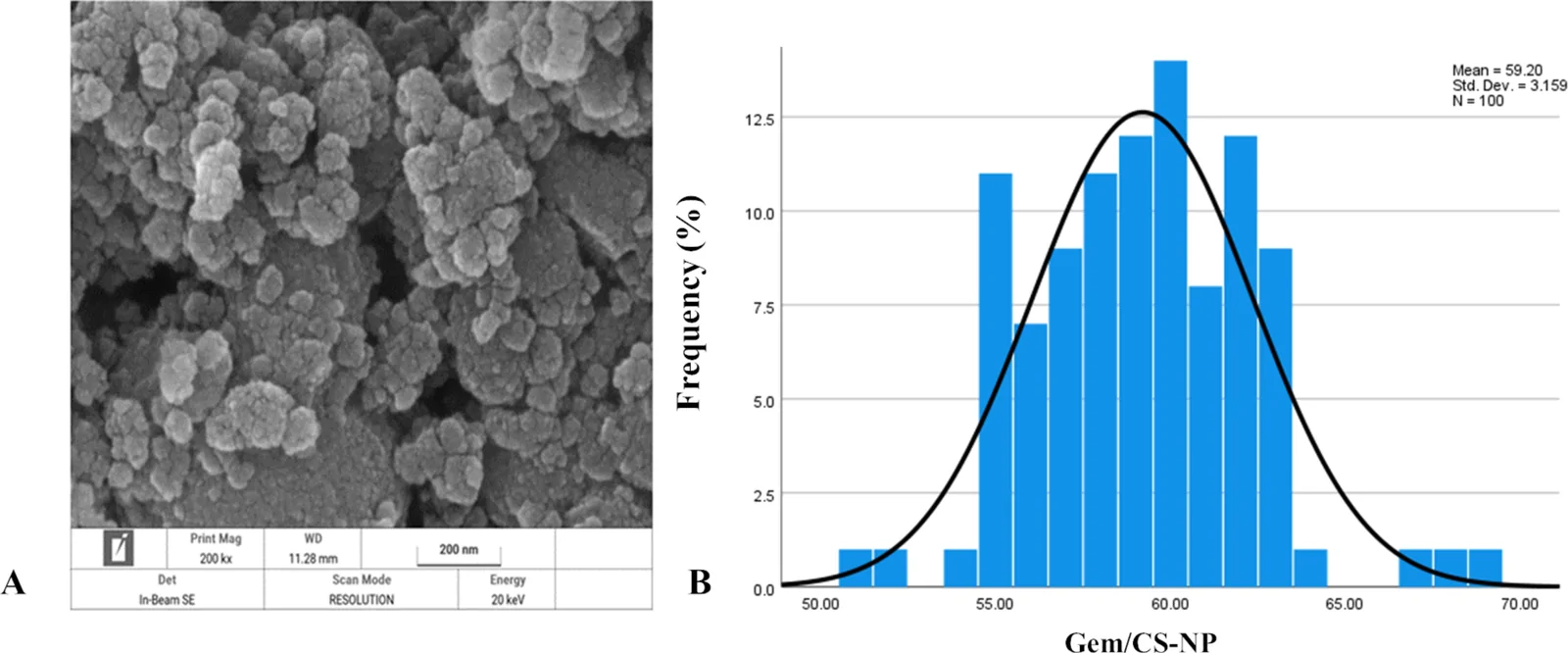
Representative FESEM micrograph of FP/Gem/CS-NPs captured at 200,000× magnification (scale bar = 200 nm; working distance = 11.28 mm) (A). Particle size distribution of FP/Gem/CS-NPs obtained by ImageJ analysis of the FESEM micrograph (*n* = 100) (B).

The morphology of the synthesized FP/Gem/CS-NPs was examined by field-emission scanning electron microscopy (FESEM) at a magnification of 200,000×, with a working distance of 11.28 mm and a scale bar of 200 nm (Figure 2A). The FESEM micrographs revealed predominantly spherical nanoparticles with a rough and granular surface morphology, which is characteristic of chitosan-based nanoparticle systems. Minor clustered structures were observed, most likely as a result of partial aggregation during sample drying and preparation for FESEM imaging rather than reflecting the dispersion state of the nanoparticles in suspension. ImageJ analysis of the FESEM micrographs showed an average particle diameter of 59.20 nm, and the corresponding particle size distribution is presented in Figure 2B. These findings confirm the successful fabrication of nanosized FP/Gem/CS-NPs with morphological characteristics consistent with chitosan-based drug delivery systems. The particle size distribution and colloidal behavior of the nanoparticles were further evaluated by DLS and zeta potential measurements.

#### Dynamic light scattering

**Figure 3:**
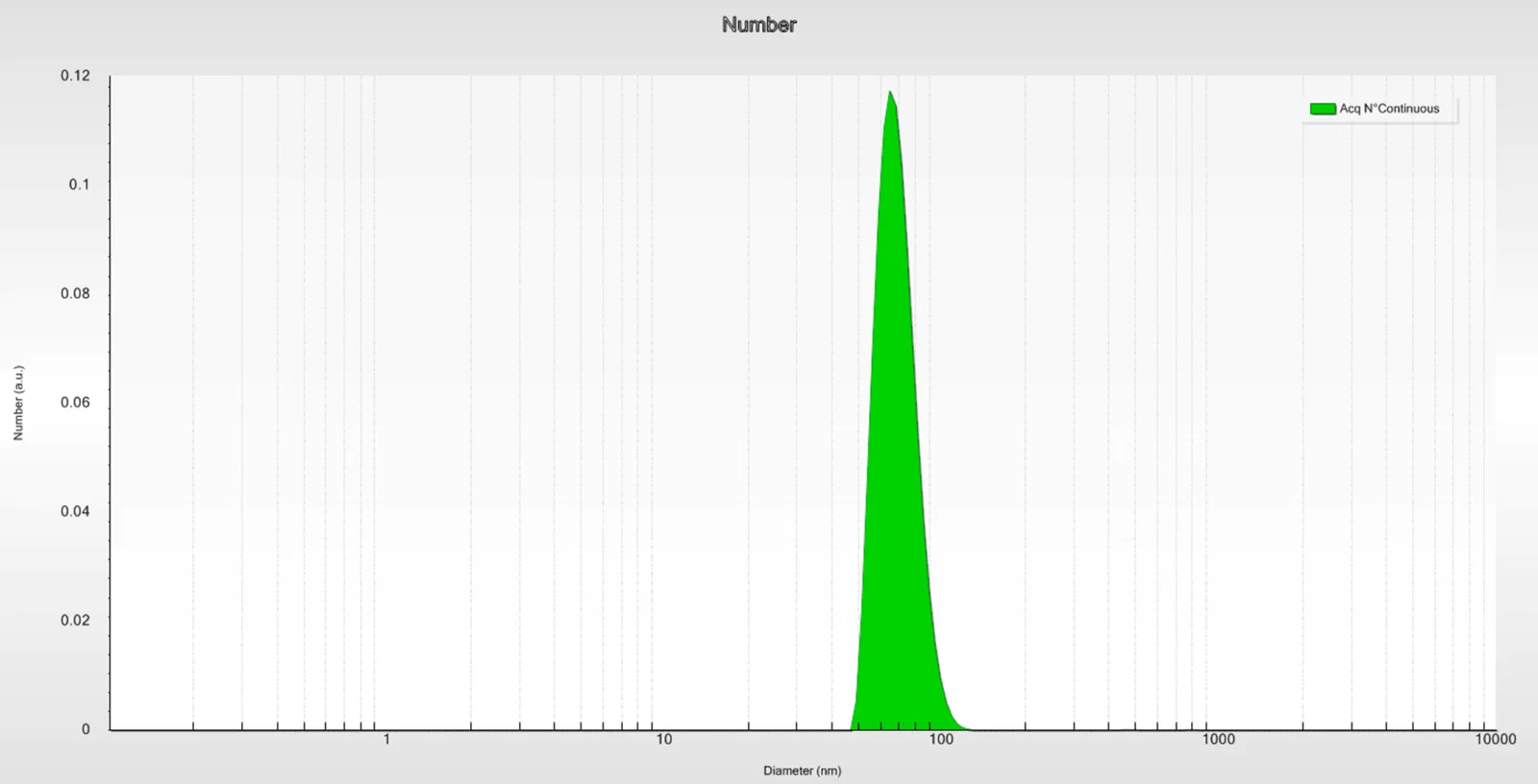
Size analysis of FP/Gem/CS-NPs using dynamic light scattering. The DLS results show a uniform particle size distribution, with sizes ranging from approximately 56 to 85 nm and an average particle size of around 69 nm. This size range is optimal for biological and pharmaceutical applications, indicating that the synthesized CS-NP possess suitable characteristics for effective drug delivery.

The particle size distribution of FP/Gem/CS-NPs was comprehensively analyzed using dynamic light scattering (DLS) at a controlled temperature of 24.99 °C in continuous acquisition mode (Figure 3). The DLS spectrum revealed a narrow, monomodal size distribution, indicating the homogeneity of the nanoparticle suspension. The mean particle diameter was measured at 68.79 nm, with a mode of 64.75 nm, and a peak intensity of 100%. These data suggests a highly uniform particle population. The FP/Gem/CS-NPs showed a mean particle size of 69.13 nm based on number distribution, with a PDI value of 0.50016, indicating a moderately broad size distribution of the nanoparticle formulation.

Further statistical analysis of the distribution highlighted that 10%, 50%, and 90% of the nanoparticles had diameters below 56.36, 67.81, and 85.45 nm, respectively. The relatively low standard deviation of 16.34% reinforces the consistency in particle size. These findings align closely with the results obtained from FESEM analysis, underscoring that the synthesized FP/Gem/CS-NPs are well within the nanometer range. This homogeneity and precise size distribution enhance their suitability for biomedical applications, particularly targeted drug delivery.

#### Zeta potential analysis

**Figure 4:**
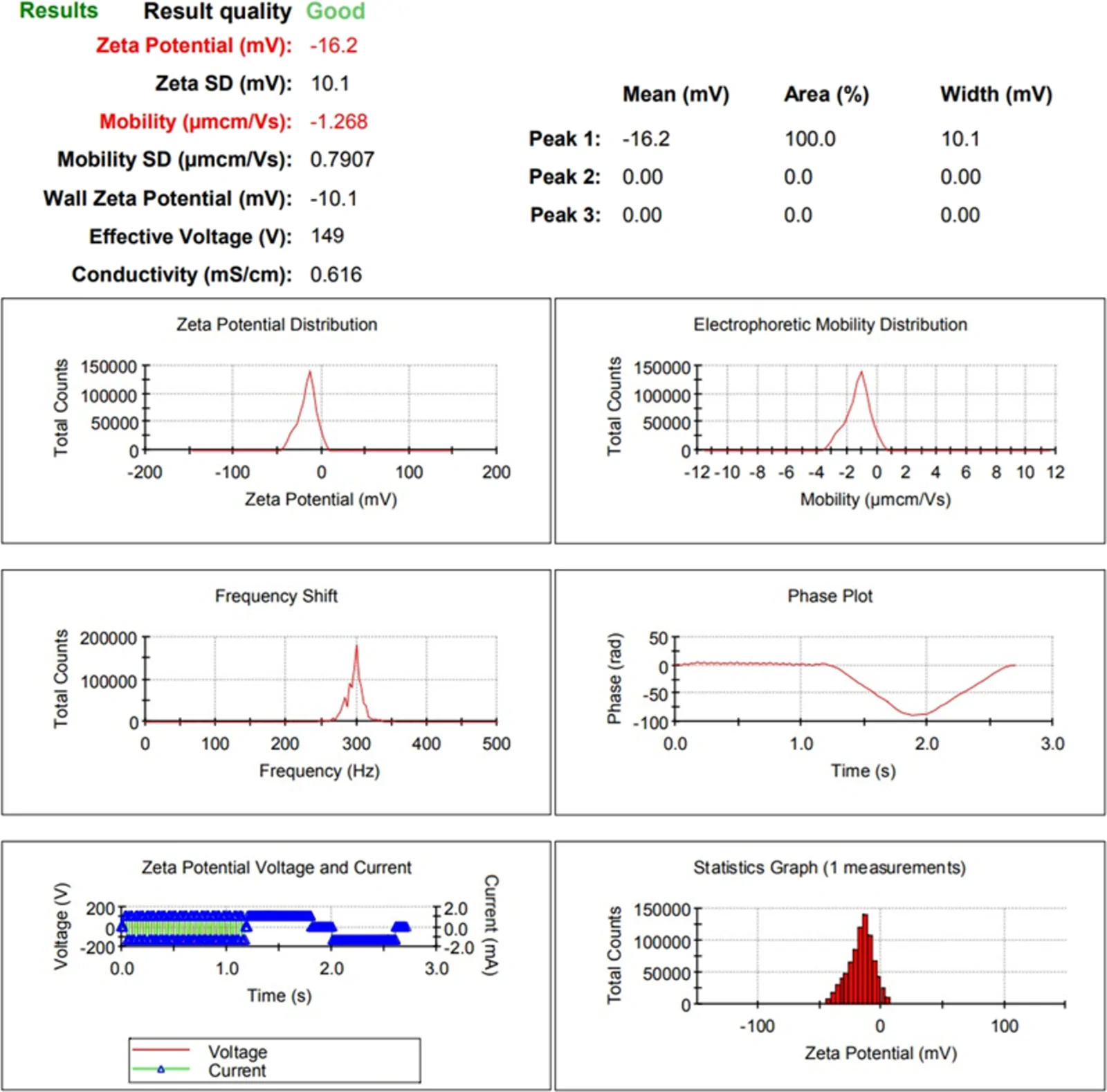
The zeta potential of FP/Gem/CS-NPs shows a single peak centered at −16.2 mV, indicating a moderately negative surface charge and good colloidal stability of the nanoparticles. Electrophoretic mobility was measured as −1.268 µm·cm/Vs. Additional plots include the electrophoretic mobility distribution, frequency shift, phase plot, voltage/current trace, and statistical histogram, confirming the uniformity and reliability of the measurement.

The surface charge of the FP/Gem/CS-NPs was evaluated by zeta potential measurements. The nanoparticles exhibited a mean zeta potential of −16.2 mV, indicating a moderately negative surface charge that contributes to electrostatic stabilization in aqueous suspension. The electrophoretic mobility was measured as −1.268 µm·cm/V·s, and the standard deviation of the zeta potential was ±10.1 mV, suggesting a relatively uniform distribution. The zeta potential distribution revealed a single peak at −16.2 mV, encompassing 100% of the measured particles, with no secondary populations detected (Figure 4). The measurement was performed at 25 °C using water as the dispersant medium, with a conductivity of 0.616 mS/cm. These findings confirm the colloidal stability of the nanoparticle formulation under physiological-like conditions.

#### Fourier-transform infrared spectroscopy

**Figure 5:**
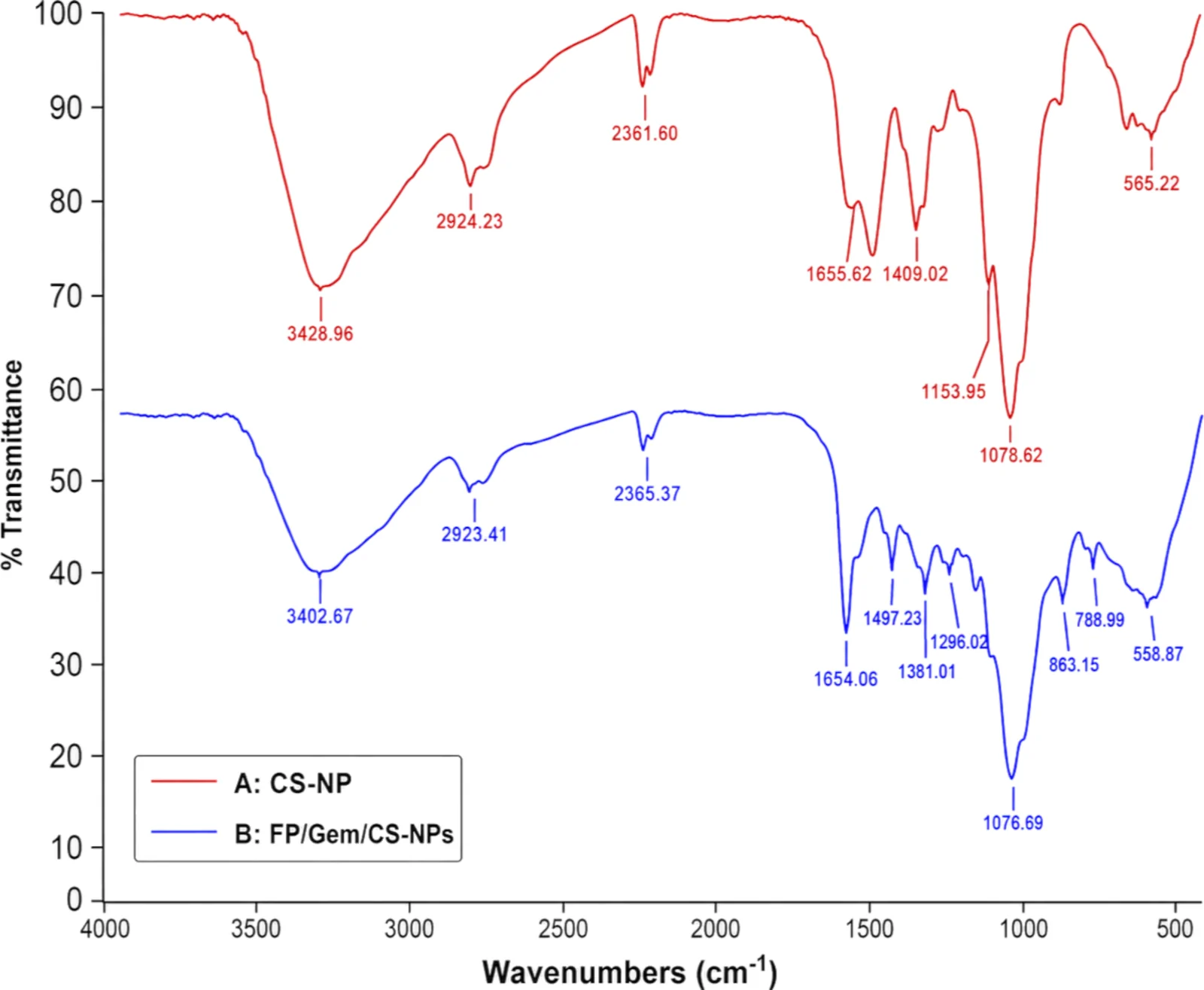
FTIR spectra of chitosan nanoparticles (CS-NPs) and *Fritillaria persica* extract/gemcitabine-loaded chitosan nanoparticles (FP/Gem/CS-NPs). The spectra were overlaid to compare the characteristic absorption bands of CS-NPs before and after loading with gemcitabine and FP extract. The observed shifts and intensity changes in the characteristic peaks indicate the successful incorporation of FP phytochemicals and gemcitabine into the chitosan nanoparticle matrix while preserving the chemical integrity of the nanoparticle components.

FTIR spectroscopy was conducted to analyze the functional groups present in the nanoparticles and to validate the successful encapsulation of Gem within the chitosan matrix (Figure 5). The resulting spectra display transmittance as a function of the wavenumber, where distinct peaks correspond to specific molecular vibrations.

In spectrum A, which represents CS-NP, a broad absorption band at 3428.95 cm^−1^ is observed, indicating O–H and N–H stretching vibrations. Peaks at 2924.23 and 2861.09 cm^−1^ are linked to C–H stretching vibrations of aliphatic chains. Additionally, peaks at 1658.62 and 1409.02 cm^−1^ signify the presence of amide I and carboxyl groups, while the peak around 1078.62 cm^−1^ corresponds to C–O stretching.

Spectrum B, representing the FP/Gem/CS-NPs, reveals similar characteristic peaks with slight shifts in wavenumbers and intensities. The peak at 3420.67 cm^−1^ indicates that the O–H/N–H bonds have been retained, while the peaks at 2929.41 and 2865.37 cm^−1^ represent C–H stretching. Notably, new or enhanced peaks at 1381.01, 1076.89, and 890.15 cm^−1^ provide strong evidence of molecular interactions between Gem and the chitosan carrier. These spectral changes collectively affirm the successful incorporation of Gem into the nanoparticle structure without any significant chemical degradation.

#### In vitro release studies

**Figure 6:**
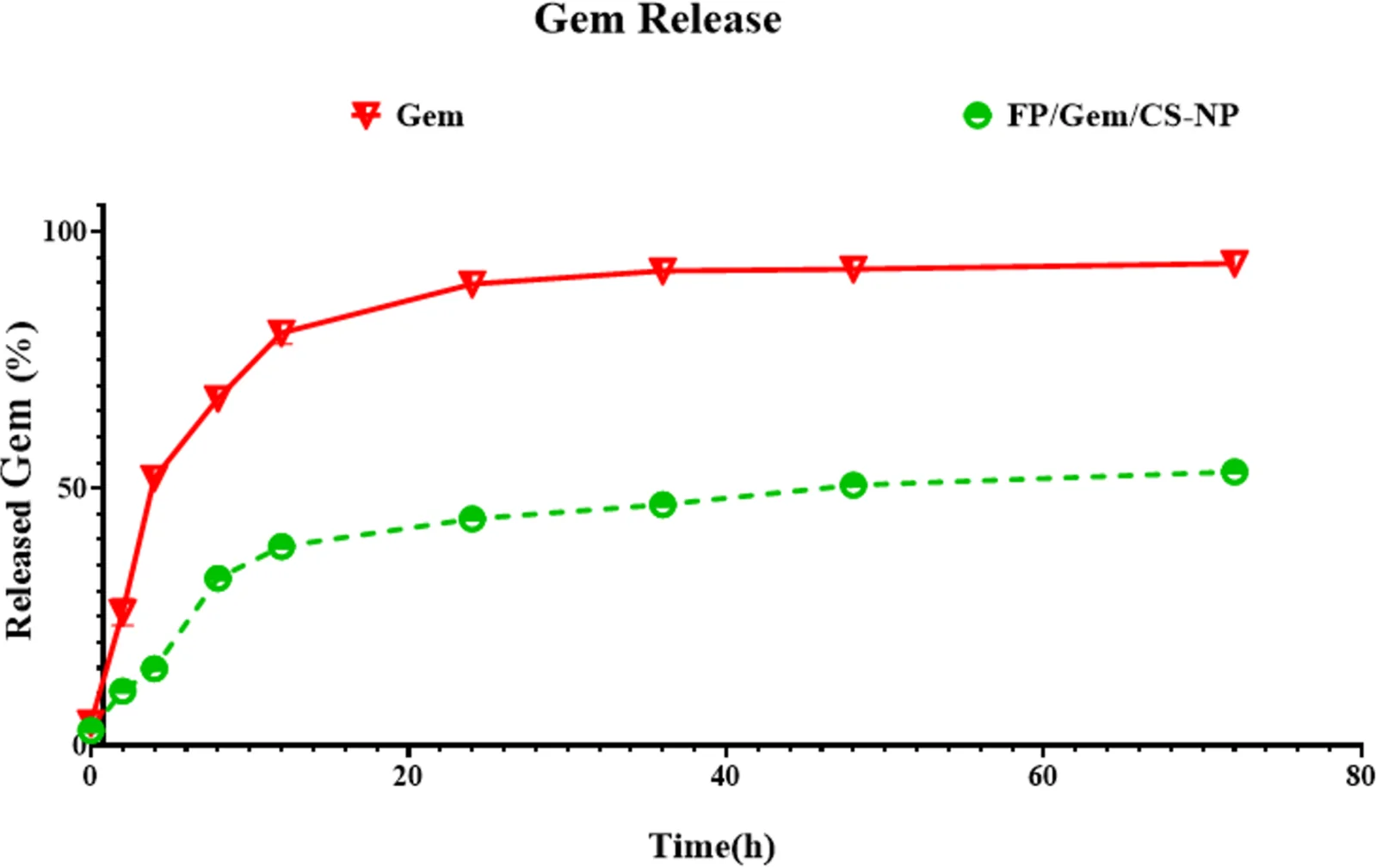
Kinetic release of free Gem and Gem loaded FP/Gem/CS-NPs in PBS at 37 °C.

The release behavior of free Gem and Gem encapsulated within FP/CS-NPs was investigated over different incubation periods. As shown in Figure 6, free gemcitabine displayed a burst release pattern, with nearly 88–91% of the drug released into the PBS medium during the first 24 h. In comparison, FP/Gem/CS-NPs exhibited a slower and more prolonged release behavior, where about 50% of the loaded gemcitabine was released gradually over 72 h. The sustained release observed for FP/Gem/CS-NPs could be associated with the diffusion of the buffer solution into the chitosan nanoparticle structure, leading to hydration and swelling of the polymer matrix followed by controlled diffusion of the entrapped drug. In addition, nanoparticle characteristics such as particle size and physicochemical properties may play important roles in regulating the release kinetics of gemcitabine from the nanoparticle system.

#### DPPH assay

**Figure 7:**
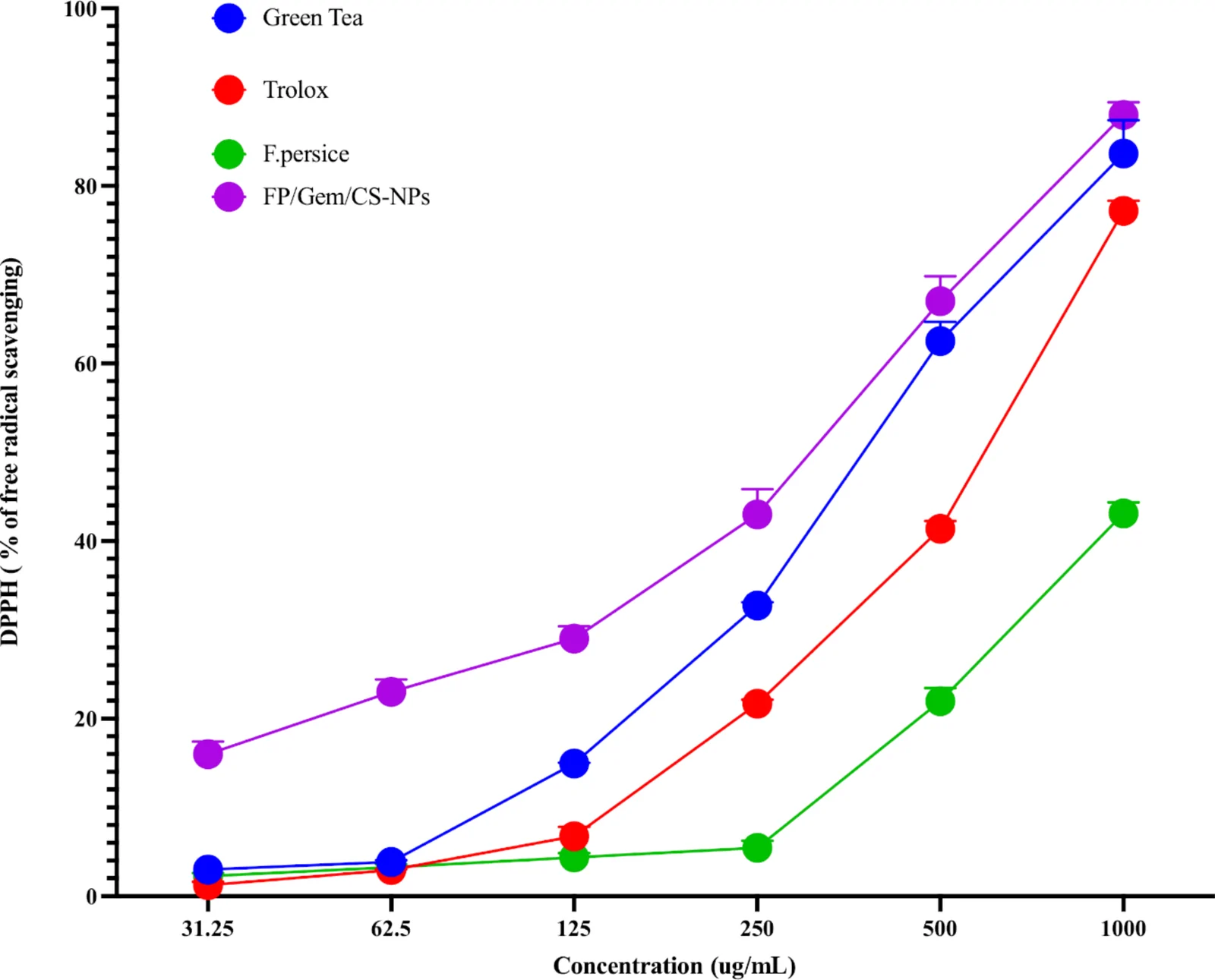
Percentage of DPPH radical scavenging activity by FP/Gem/CS-NPs compared to the standard antioxidants.

The antioxidant capacity of FP/Gem/CS-NPs was assessed by measuring their DPPH radical scavenging activity, which is a valuable method for evaluating antioxidant effectiveness. This study compared FP/Gem/CS-NPs with the standard antioxidant Trolox and green tea (serving as a control) at concentrations ranging from 0 to 1200 µg/mL. The results presented in Figure 7 reveal that the IC_50_ values for FP and FP/Gem/CS-NPs were 1196.08 and 254.228 µg/mL, respectively. These findings highlight the significantly more substantial antioxidant potential of FP/Gem/CS-NPs compared to FP indicating their promising role in antioxidant applications.

#### MTT assay

The evaluation of cytotoxic effects from FP extract, Gem, and the combination formulation of FP/Gem/CS-NPs was conducted in two cancer cell lines at varying concentrations (0–500 µg/mL) over 24 and 48 h using MTT assays. The results indicate a promising dose- and time-dependent reduction in cell viability across all treatment groups. Notably, the FP/Gem/CS-NPs formulation consistently showed the most significant cytotoxic impact compared to FP or Gem alone, suggesting that the combination enhances effectiveness. At 24 and 48 h, the nanoparticle treatment significantly decreased cell viability at concentrations above 100 µg/mL, with more pronounced effects observed at the 48 h mark. While Gem displayed moderate cytotoxicity and FP had relatively lower activity, their integration within the nanoparticle system indicates a potential synergy that enhances therapeutic efficacy. These findings highlight the promise of FP/Gem/CS-NPs as an effective anticancer strategy, which can achieve improved potency compared to single-agent treatments. This underscores the importance of continued research into combination therapies for better cancer treatment outcomes.

**Figure 8:**
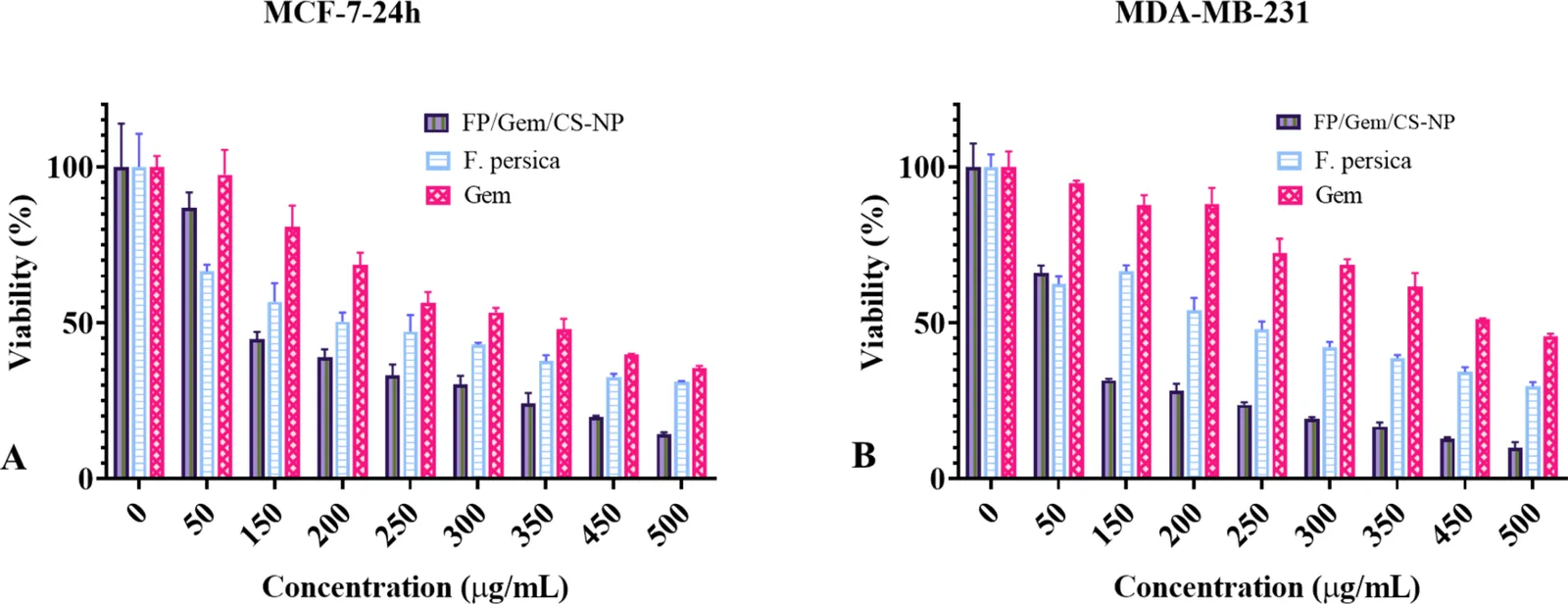
Cell viability assessment by MTT method. MCF-7(A) and MDA-MB-231(B) cell lines were treated with FP/Gem/CS-NP, FP extract, and Gem at increasing concentrations (0–500 µg/mL) for 24 and 48 h, respectively. The survival rate of cells decreased in a dose- and time-dependent manner, with the FP/Gem/CS-NPs treatment exhibiting significantly greater cytotoxicity compared to FP or Gem alone in both cell lines. All experiments were performed in triplicate, and data are presented as mean ± SD.

Both cell lines were treated with FP extract, Gem, and a combination of both with NPs. Viability and IC_50_ were evaluated using the MTT method. The IC_50_ values from treatment with the FP/Gem/CS-NPs combination for MCF-7 and MDA-MB-231 cell lines were calculated. The results showed that simultaneous treatment with NPs containing FP extract and gem limited the viability of breast cancer cell lines compared to when they were used alone (Figure 8 and Table 2).

**Table 2:** The IC_50_ values (µg/mL) of different treatments on MCF-7 and MDA-MB-231 breast cancer lines after 24 and 48 h.

Cell Line	Treatment	IC_50_ (24 h)	IC_50_ (48 h)

MDA-MB-231	FP/Gem/CS-NP	85.98	78.09
	FP	199.7	126.7
	Gem	392.6	288.0

MCF-7	FP/Gem/CS-NP	150.5	76.05
	FP	182.4	77.37
	Gem	331.1	278.0

#### Caspase-3/7 activity

**Figure 9:**
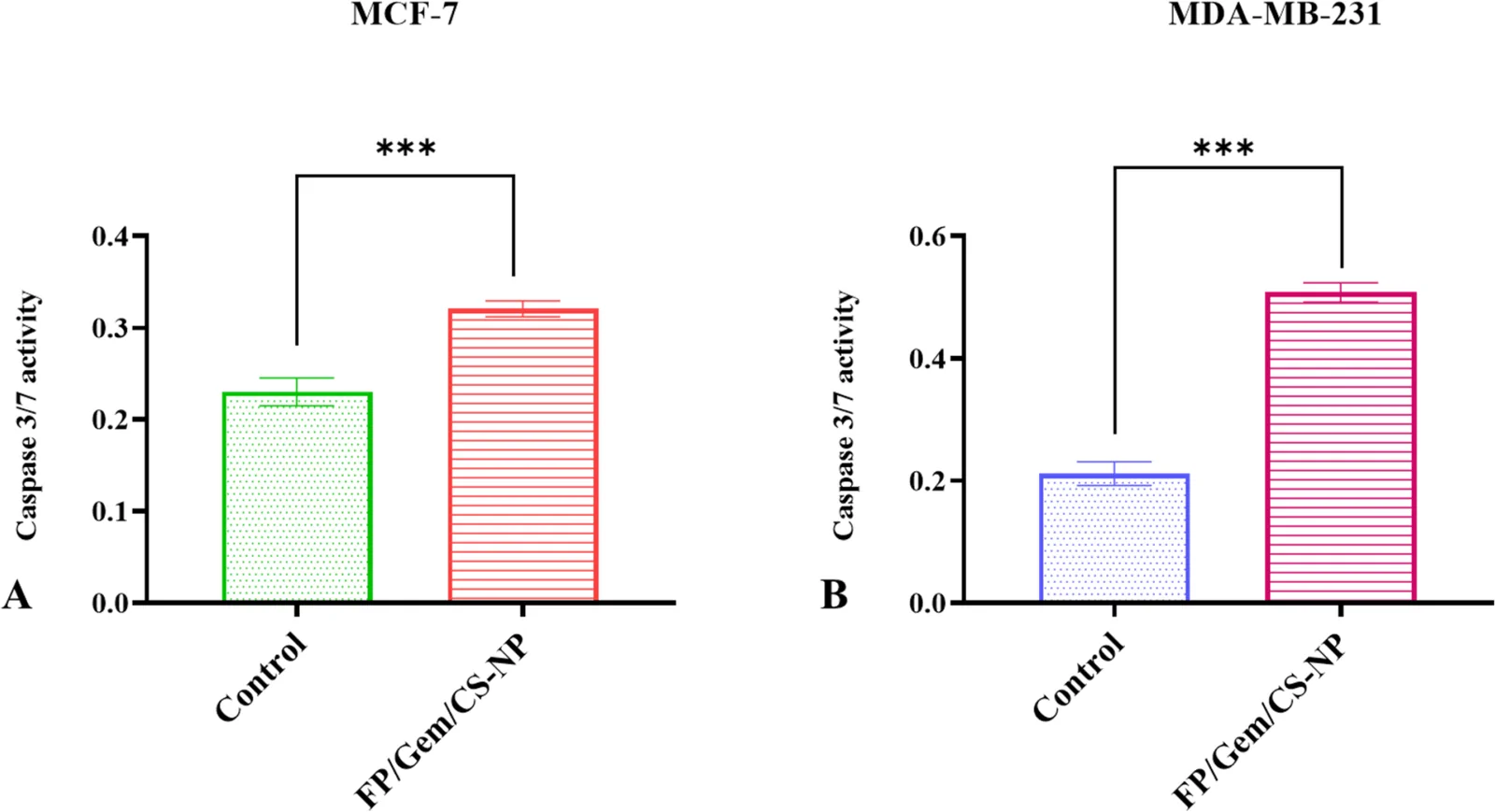
Caspase-3/7 activity in breast cancer cell lines following treatment with FP/Gem/CS-NP. (A) MCF-7 and (B) MDA-MB-231 cells were analyzed for relative caspase-3/7 activity after FP/Gem/CS-NPs exposure. A significant increase in caspase activity was observed in both cell lines compared to the untreated control group, indicating apoptosis induction. All experiments were performed in triplicate (* indicates *P* < 0.05, ** indicates *P* < 0.01, *** indicates *P* < 0.001, ns indicates non-significance).

The activation of caspase-3/7, a key marker of apoptosis, was evaluated in MCF-7 and MDA-MB-231 breast cancer cells following treatment with FP/Gem/CS-NP. As illustrated in Figure 9A, the treatment led to a significant increase in caspase-3/7 activity in MCF-7 cells compared to the control group (*P* < 0.001), demonstrating a successful induction of apoptosis. In Figure 9B, MDA-MB-231 cells showed an even more substantial elevation in caspase-3/7 activity (*P* < 0.0001), indicating a stronger apoptotic response in this more aggressive and invasive triple-negative breast cancer cell line.

These findings significantly highlight the pro-apoptotic potential of the FP/Gem/CS-NPs formulation in both estrogen receptor-positive (MCF-7) and triple-negative (MDA-MB-231) breast cancer models. The increased caspase-3/7 activity suggests that the cytotoxic effects observed in previous viability assays are primarily mediated through caspase-dependent apoptotic pathways. Collectively, these data support the conclusion that FP/Gem/CS-NPs effectively trigger apoptosis, positioning them as a promising nanotherapeutic strategy for breast cancer treatment. This approach could pave the way for more effective treatment options, emphasizing the importance of further exploration in clinical applications.

#### Real-time PCR

**Figure 10:**
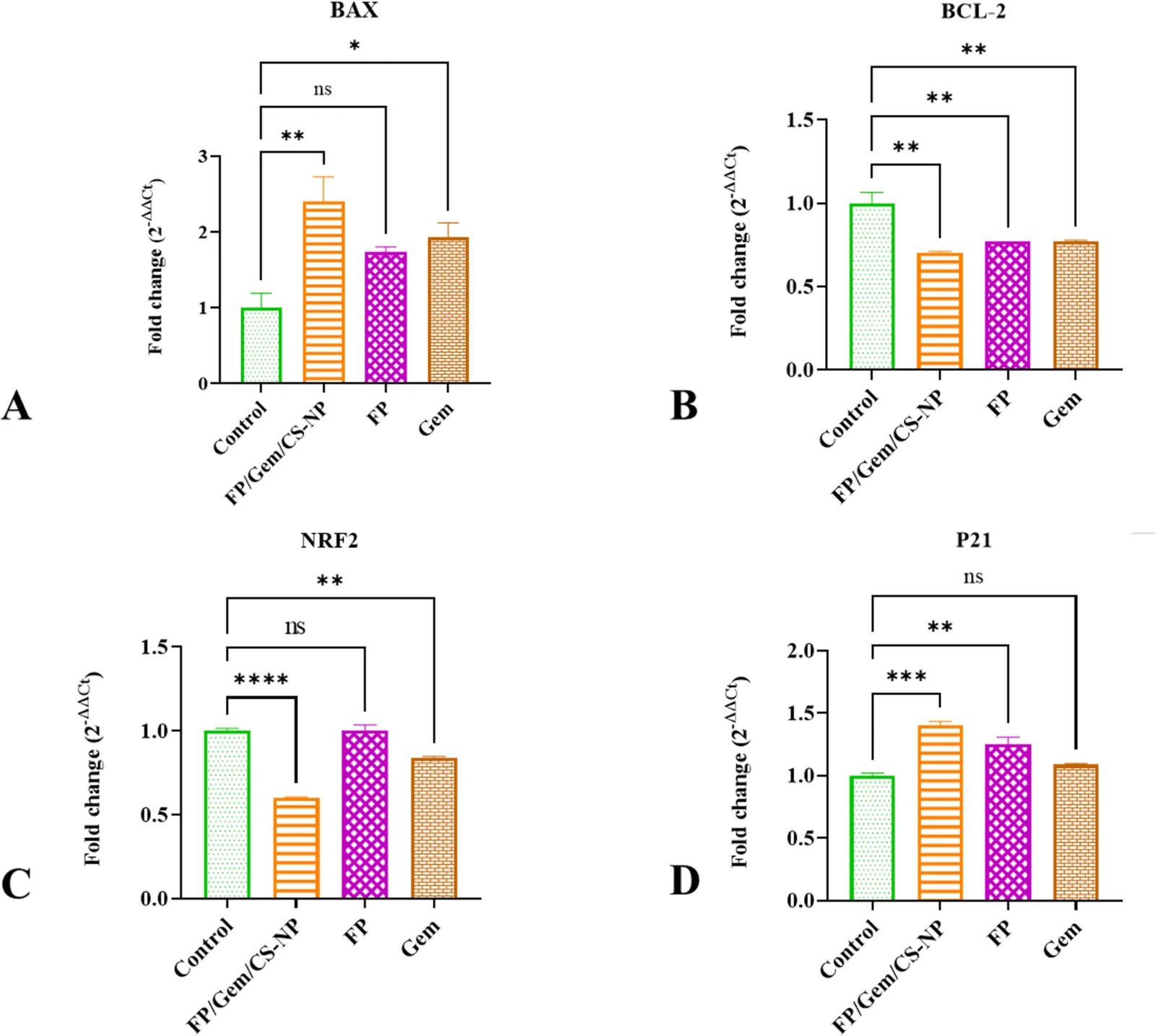
Expression of (A) BAX, (B) BCL-2, (C) NRF2, and (D) P21 genes in the MCF-7 cell line. Gene expression analysis revealed that treatment with FP/Gem/CS-NPs significantly altered the expression profile of key regulatory genes involved in apoptosis, oxidative stress response, and cell cycle control. Specifically, the FP/Gem/CS-NPs group exhibited a marked increase in BAX and P21 expression and a significant decrease in BCL-2, indicating a shift toward pro-apoptotic signaling. NRF2 expression was also elevated, suggesting activation of antioxidant defense pathways. These findings confirm that the treatment disrupts the gene expression balance in favor of apoptosis induction. All experiments were performed in duplicate (* indicates *P* < 0.05, ** indicates *P* < 0.01, *** indicates *P* < 0.001, ns indicates non-significance).

The gene expression analysis comprehensively examined four experimental groups, that is, the untreated control, FP/Gem/CS-NP, FP, and Gem. In the MCF-7 cell line, treatment with FP/Gem/CS-NPs and Gem notably enhanced the expression of the pro-apoptotic BAX gene (Figure 10A), highlighting the potential of these treatments to initiate apoptosis. Interestingly, the FP treatment alone did not yield significant changes in BAX expression, suggesting that its pro-apoptotic effects may depend on the presence of Gem or the nanocarrier. Furthermore, the analysis revealed that the expression of the anti-apoptotic gene BCL-2 (Figure 10B) was significantly decreased in all three treatment groups compared to the control. This reduction points to a favorable shift in the balance of apoptotic signals, further supporting a move toward apoptosis in the cancer cells. Additionally, the expression of NRF2 (Figure 10C), which is critical for oxidative stress regulation, was significantly increased in the FP/Gem/CS-NPs and Gem groups, underscoring their ability to enhance cellular defenses against oxidative stress. However, changes in NRF2 expression were not observed in the FP group, indicating that the combination treatments may be more effective in this regard. Moreover, the P21 gene, associated with cell cycle arrest, exhibited significant upregulation following treatments with FP/Gem/CS-NPs and FP (Figure 9D). Gem treatment did not cause notable changes in P21 expression. This suggests that FP, especially in combination with Gem, may play a role in inducing cell cycle arrest, thus further contributing to anti-cancer effects.

**Figure 11:**
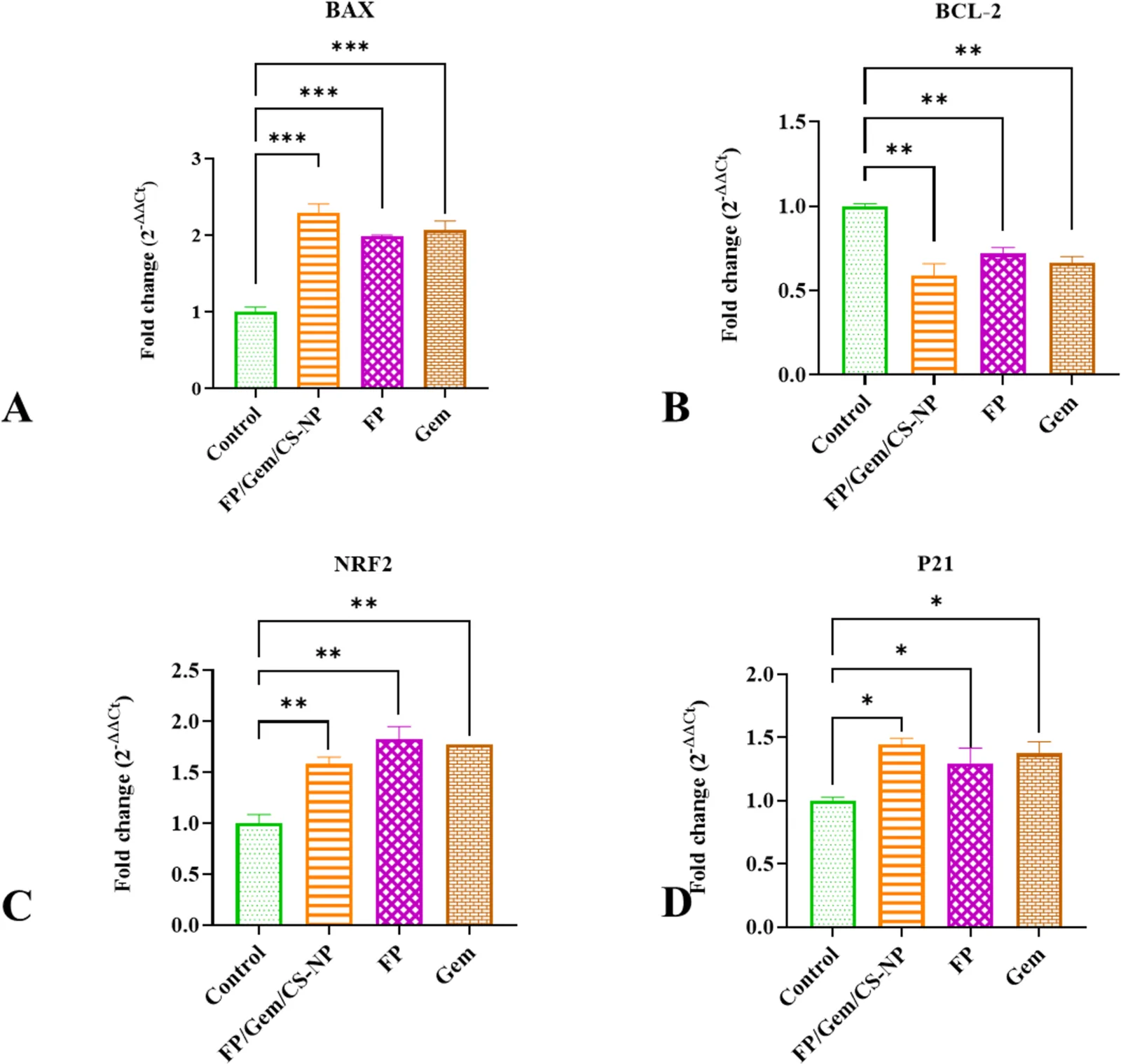
Relative expression levels of (A) BAX, (B) BCL-2, (C) NRF2, and (D) P21 genes in the MDA-MB-231 cell line. Examination of relative expression data in the MDA-MB-231 cell line revealed that FP/Gem/CS-NPs treatment modulates the genes studied to induce apoptosis. All experimental groups were examined in duplicate (* indicates *P* < 0.05, ** indicates *P* < 0.01, *** indicates *P* < 0.001, ns indicates non-significance).

The treatment strategies yielded even broader effects in the more aggressive MDA-MB-231 cell line. The expression levels of BAX, NRF2, and P21 (Figure 11A–C) were significantly increased across all treatment groups (FP/Gem/CS-NP, FP, and Gem) compared to the untreated control. This indicates a robust response to these treatments, regardless of the specific intervention. Additionally, BCL-2 expression (Figure 11D) was consistently downregulated across all treatment groups, reinforcing the pro-apoptotic and anti-survival effects of the applied therapies.

These findings suggest that while MCF-7 cells respond selectively to treatment conditions, the MDA-MB-231 cells demonstrate a more comprehensive and uniform gene expression response. Notably, the FP/Gem/CS-NPs treatment appears to consistently and significantly modulate key apoptotic and regulatory genes in both cell lines, emphasizing its potential as a valuable therapeutic approach in the fight against breast cancer. These insights lay the groundwork for further research into optimizing treatment strategies that could enhance therapeutic efficacy in breast cancer management. Fold change of genes are shown in Table 3.

**Table 3:** Fold change of gene expression of BAX, BCL-2, NRF2, and P21 genes in the MCF-7 and MDA-MB-231 cell line (↑ indicates upregulation, ↓ indicates down-regulation, NS indicates non-significance).

	Gene name	FP/Gem/cs-N	FP	Gem

MCF-7	BAX	2.39792 ↑	1.74161 NS	1.93606 ↑
	BCL-2	1.41908 ↓	1.29684 ↓	1.29686 ↓
	NRE	1.65863 ↓	1.00029 NS	1.1933 ↓
	P21	1.3998 ↑	1.25327 ↑	1.08676 NS

MDA-MB-231	BAX	2.28319 ↑	1.97936 ↑	2.07204 ↑
	BCL-2	1.693506 ↓	1.389083 ↓	1.504153 ↓
	NRE	1.58085 ↑	1.82348 ↑	1.77154 ↑
	P21	1.44435 ↑	1.29516 ↑	1.3724 ↑

#### Apoptosis

**Figure 12:**
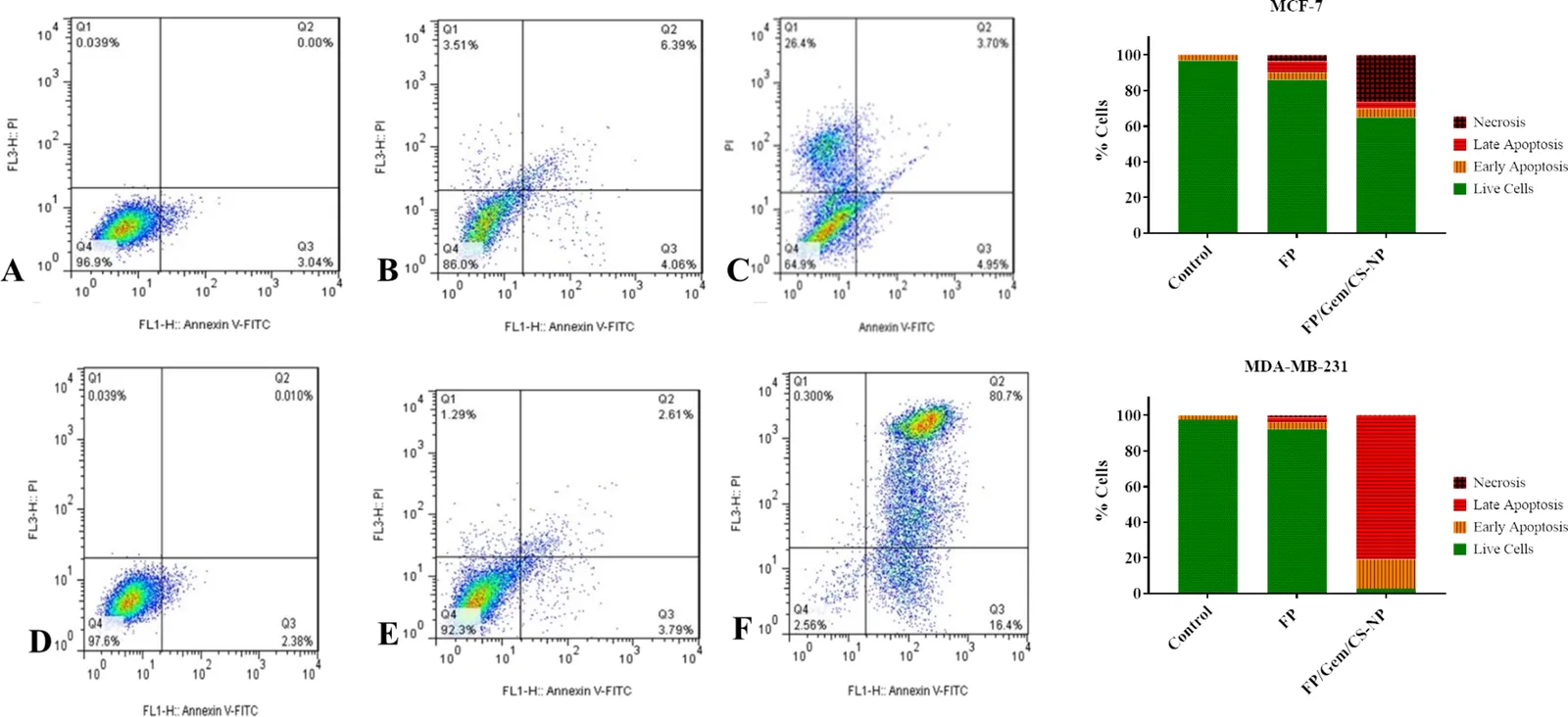
Flow cytometry assessed the Apoptosis rate in MCF-7 and MDA-MB-231 cell lines. Panels A–C represent MCF-7 cells, while panels D–F correspond to MDA-MB-231 cells. (A, D) Untreated control groups showed minimal levels of apoptosis. (B, E) Cells treated with FP extract exhibited a moderate increase in apoptotic population. (C, F) Treatment with FP/Gem/CS-NPs led to a substantial elevation in apoptosis rates in both cell lines, indicating a strong pro-apoptotic effect of the nanoparticle formulation.

In this study, we employed flow cytometry to effectively evaluate apoptosis and necrosis levels in different cell lines. In the MCF-7 cell line, the untreated group exhibited an impressive cell viability rate of 96.9%, with only 3.04% of the cells undergoing apoptosis and minimal necrosis observed. However, in the group treated with the FP/Gem/CS-NPs for 48 h, we noted a significant decrease in viable cells to 64.9%, accompanied by approximately 35% of the cells entering the apoptosis stage. In the MDA-MB-231 cell line, the untreated group primarily consisted of viable cells, showing only slight signs of apoptosis or necrosis. This control group provides a valuable baseline for comparison with the treated groups. The treatment with FP/Gem/CS-NPs distinctly enhanced both primary and secondary apoptosis and necrosis, highlighting the compound’s potential toxic or inhibitory effects on cell viability. As expected, and corroborated by prior research, Gem, a proven chemotherapy agent, effectively induces apoptosis and reduces overall cell survival. The results illustrated in Figure 12 indicate that treatment with the FP extract alone has a mild effect on apoptosis and necrosis. These findings suggest that when used independently, the FP extract demonstrates less protective or toxic effects compared to its combination with Gem and CS-NP, offering important insights for future research and therapeutic strategies.

#### Cell cycle analysis

**Figure 13:**
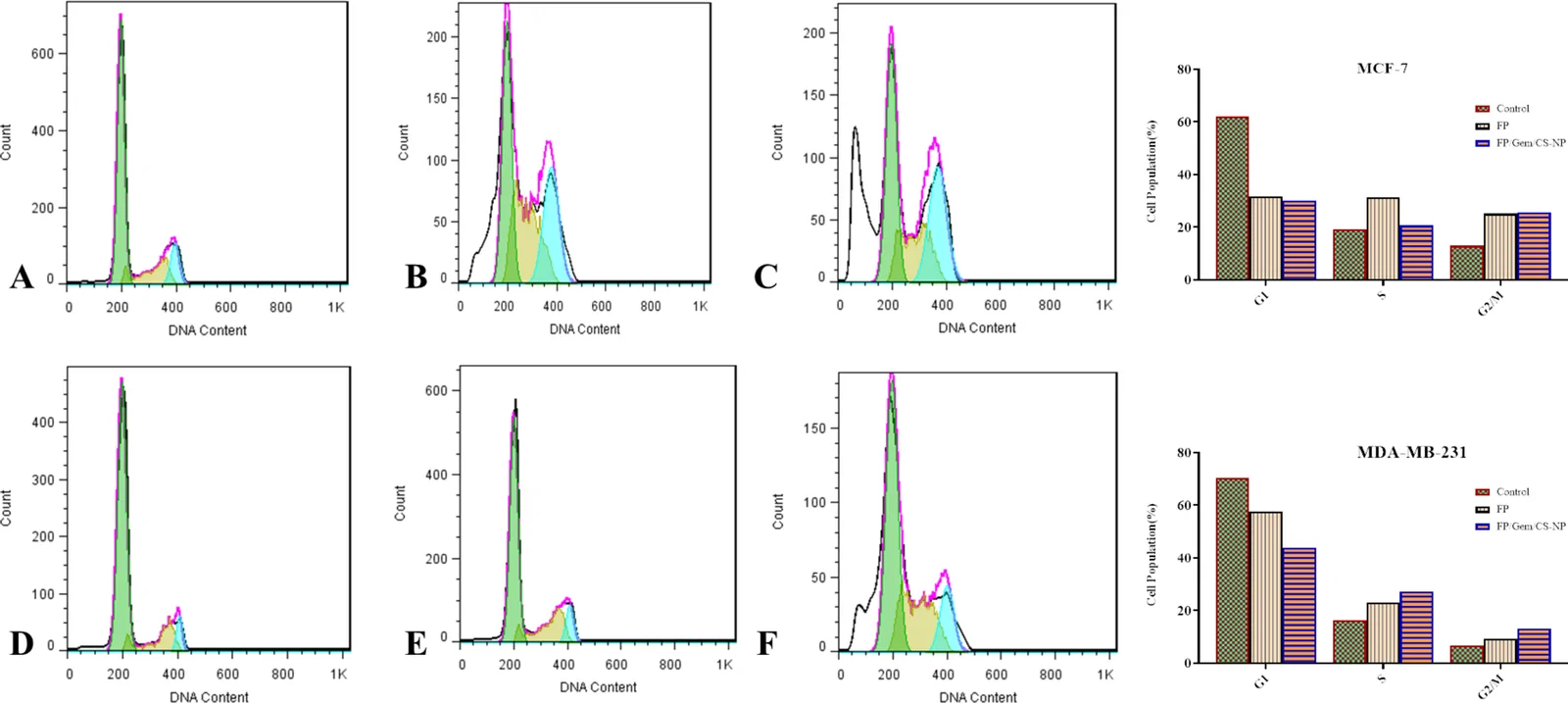
Flow cytometry assessed the cell cycle analysis in MCF-7 and MDA-MB-231 cell lines. Panels A–C represent MCF-7 cells, while panels D–F correspond to MDA-MB-231 cells.

The cell cycle results showed that in the MCF-7 cell line, after treatment with FP and FP/Gem/CS-NP, the cell frequency in the G1 phase was sharply reduced. In untreated cells, the cell frequency was 62.06%, and after treatment with FP and FP/Gem/CS-NP, it reached 31.71% and 30.15%, respectively. However, the cell frequency in the G2M phase increased. In the MDA-MB-231 cell line, after treatment with FP and FP/Gem/CS-NP, the cell frequency in the G1 phase was sharply reduced. In untreated cells, the cell frequency was 70.47%, and after treatment with FP and FP/Gem/CS-NP, it reached 57.55 and 47.87%, respectively. However, the cell frequency in the G2M phase increased. Figure 13 shows the details of cell cycle analysis of MCF-7 and MDA-MB-231 cells before and after FP and FP/Gem/CS-NPs treatment.

## Discussion

Optimizing the efficacy of chemotherapeutic agents while minimizing systemic toxicity presents a significant challenge in the management of breast cancer. This study introduces a novel nanotherapeutic formulation that combines Gem, a standard cytotoxic agent, with FP extract encapsulated within CS-NPs. The primary objective of this combination is to enhance anticancer efficacy through synergistic interactions while mitigating the adverse effects typically associated with Gem. While Gem is effective in treating breast cancer, it is known to induce oxidative stress and inflammation, which can lead to off-target toxicity [31]. FP, a medicinal plant recognized for its antioxidant and anti-inflammatory properties, may counteract these deleterious effects [32]. The co-delivery of Gem and FP using CS-NPs provides several therapeutic advantages, including enhanced drug stability, targeted delivery, and improved cellular uptake. The physicochemical characteristics of the nanoparticles, such as their size (≈69 nm), morphology, and uniform dispersion, underscore their suitability for tumor penetration and retention, both of which are critical in oncological nanomedicine. The sustained release profile of gemcitabine from FP/Gem/CS-NPs can be attributed to the physicochemical characteristics of the nanoparticle formulation. The nanoscale particle size provides a relatively large surface area, allowing for an initial diffusion of gemcitabine molecules located near or at the nanoparticle surface, which contributes to the early release phase. Subsequently, the crosslinked chitosan matrix limits drug diffusion by acting as a polymeric barrier, leading to a slower and more sustained release. In addition, the hydrophilic nature of chitosan and its intermolecular interactions with gemcitabine may further retard drug diffusion. Therefore, the release behavior is governed by a combination of diffusion through the hydrated polymer matrix and the structural properties of the nanoparticles.

In vitro assays conducted using MCF-7 and MDA-MB-231 breast cancer cell lines demonstrated that the FP/Gem/CS-NPs formulation significantly reduced cell viability in a dose- and time-dependent manner, surpassing the effects of either agent administered alone [33–35]. Apoptosis assays, including caspase-3/7 activity analysis and flow cytometry, confirmed that the combination treatment enhanced programmed cell death [36]. These results suggest a synergistic interaction between Gem and FP facilitated by the nanoparticle carrier. Gene expression analyses provided additional insights into the underlying mechanisms. The pro-apoptotic gene BAX was significantly upregulated, whereas the anti-apoptotic gene BCL-2 was downregulated, indicating activation of the intrinsic apoptotic pathway [37]. Notably, the upregulation of NRF2 suggests activation of antioxidant defense mechanisms, potentially in response to Gem-induced oxidative stress. Furthermore, increased expression of P21 implies that the combination treatment may also induce cell cycle arrest, thereby contributing to its antiproliferative effects [38]. These findings align with previous studies reporting enhanced therapeutic outcomes when Gem is delivered via nanoparticle systems [39–41]. The incorporation of FP further strengthens this strategy by reducing inflammation and improving tumor selectivity. Collectively, these data underscore the therapeutic potential of this formulation in overcoming the limitations associated with conventional chemotherapy.

Despite the promising nature of these findings, this study is limited to in vitro analysis. Further preclinical investigations are essential to evaluate in vivo efficacy, pharmacokinetics, biodistribution, and potential immune interactions. Such studies are necessary to establish safety profiles and therapeutic indices before clinical translation.

The observed alterations in gene expression provide critical insights into the molecular mechanisms underlying the therapeutic effects of FP/Gem/CS-NPs. The upregulation of BAX, coupled with the downregulation of BCL-2, strongly supports activation of the intrinsic (mitochondrial) apoptotic pathway. This pathway involves mitochondrial outer membrane permeabilization, leading to the release of cytochrome c and subsequent activation of caspase-3/7, as confirmed by our functional assays. Simultaneously, the upregulation of NRF2 indicates activation of the NRF2-ARE signaling pathway, a key cellular defense mechanism against oxidative stress. This response is likely triggered by increased levels of reactive oxygen species induced by Gem exposure. Additionally, elevated expression of P21 points to activation of the p53–P21 axis, a central regulator of cell cycle arrest in response to DNA damage and cellular stress. Together, these findings suggest a dual-action mechanism of FP/Gem/CS-NPs involving the promotion of mitochondrial-mediated apoptosis alongside enhancement of the antioxidant defense system. This coordinated modulation of apoptotic and oxidative stress-related pathways likely underlies the enhanced cytotoxicity and therapeutic efficacy observed in treated breast cancer cell lines (MCF-7 and MDA-MB-231). These gene-level alterations establish a clear mechanistic link between FP/Gem/CS-NPs treatment and key signaling cascades, including mitochondrial apoptosis, NRF2-ARE, and p53–P21 pathways, thereby advancing our understanding of its molecular mode of action and reinforcing its potential as a synergistic nanotherapeutic platform for breast cancer treatment.

In addition, although the physicochemical characterization of FP/Gem/CS-NPs was performed using DLS, FESEM, FTIR, zeta potential analysis, and in vitro drug release studies, a direct comparative analysis between unloaded CS-NPs and FP/Gem/CS-NPs was not conducted in the present study. Furthermore, TEM analysis was not performed due to technical limitations. Future studies should include comprehensive comparative characterization of unloaded and loaded nanoparticles using TEM, DLS, and surface charge analysis to better elucidate the structural changes induced by FP and Gem incorporation and to further validate nanoparticle stability.

## Conclusion

The findings of this study demonstrate that the combination of Gem- and FP extract-loaded chitosan nanoparticles (CS-NPs) offers a promising therapeutic approach for breast cancer treatment. This formulation enhances the anticancer efficacy of conventional chemotherapy while potentially reducing its associated side effects. The combination therapy induced apoptosis and inhibited cancer cell proliferation in both MCF-7 and MDA-MB-231 cell lines. Characterization techniques, including FTIR and DLS, confirmed the high quality and biologically suitable size of the synthesized nanoparticles. Molecular analyses further validated the therapeutic effects by demonstrating upregulation of the pro-apoptotic gene BAX and downregulation of the anti-apoptotic gene BCL-2, indicating activation of apoptotic pathways. Given these encouraging findings, FP/Gem/CS-NPs may serve as a valuable and less toxic therapeutic alternative for breast cancer treatment. However, additional studies, particularly in vivo investigations, are necessary to fully evaluate the safety, efficacy, and clinical potential of this nanotherapeutic strategy.

## Data Availability

All data that supports the findings of this study is available in the published article and/or the supporting information of this article.
